# Supplementary material for: The causal role of circulating inflammatory markers in osteoporosis: a bidirectional Mendelian randomized study
Source: Front Immunol. 2024 Jul 18;15:1412298. doi: 10.3389/fimmu.2024.1412298 (PMC11291241; doi:10.3389/fimmu.2024.1412298)
Supplement: Supplementary file 1 [file DataSheet_1.zip › supplementary material/CIM-OSTEOPOROSIS /GCST90274816.csv_leave_one_out.pdf]

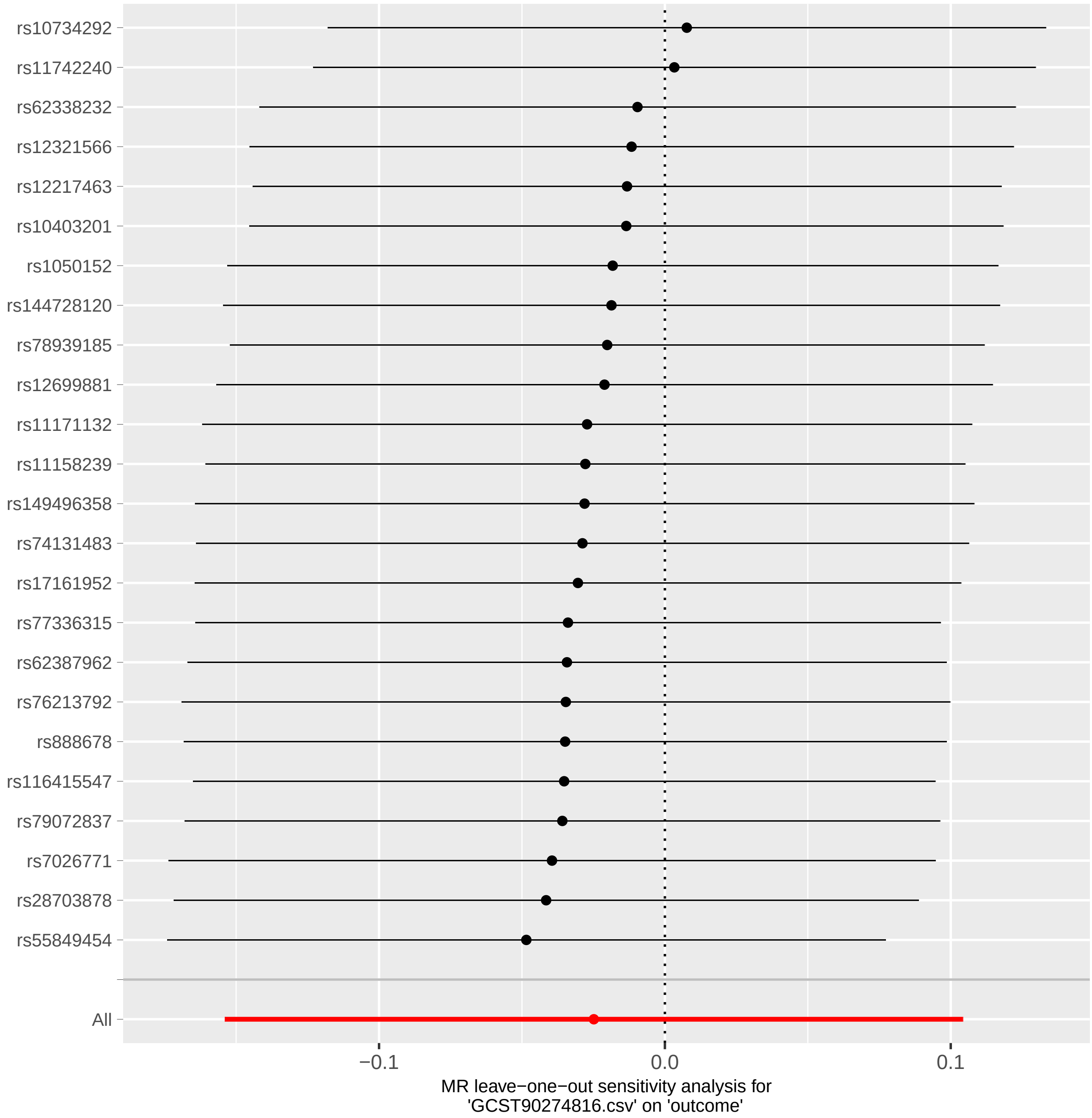
rs10734292
rs11742240
rs62338232
rs12321566
rs12217463
rs10403201
rs1050152
rs144728120
rs78939185
rs12699881
rs11171132
rs11158239
rs149496358
rs74131483
rs17161952
rs77336315
rs62387962
rs76213792
rs888678
rs116415547
rs79072837
rs7026771
rs28703878
rs55849454
All
−0.1
0.0
0.1
MR leave−one−out sensitivity analysis for
'GCST90274816.csv' on 'outcome'
